# Correction: Relationship of spirituality, health engagement, health belief and attitudes toward acceptance and willingness to pay for a COVID-19 vaccine

**DOI:** 10.1371/journal.pone.0315519

**Published:** 2024-12-05

**Authors:** Sri Handayani, Yohanes Andy Rias, Maria Dyah Kurniasari, Ratna Agustin, Yafi Sabila Rosyad, Ya Wen Shih, Ching Wen Chang, Hsiu Ting Tsai

There are errors in the Funding statement. The correct Funding statement is as follows: This research was funded by the Ministry of Science and Technology (MOST), Taiwan, through grant nos. MOST 106-2314-B-038-013-MY3, MOST 109-2314-B-038-110-, and MOST 110-2314-B-038-081-My3 by H.T.T. The funders had no role in study design, data collection and analysis, decision to publish, or preparation of the manuscript.
