# Supplementary material for: Relationship of spirituality, health engagement, health belief and attitudes toward acceptance and willingness to pay for a COVID-19 vaccine
Source: PLoS One. 2022 Oct 12;17(10):e0274972. doi: 10.1371/journal.pone.0274972 (PMC9555617; doi:10.1371/journal.pone.0274972)
Supplement: S1 Table — (DOCX) [file pone.0274972.s003.docx]

**S1 Table. Comparisons of Citizen’s Spirituality, Health Engagement, and Attitudes with Their Acceptance and Willingness to Pay for the COVID-19 Vaccine (*n*=1423)**

| **Variables** | **All participants** | **Acceptance** | | **Willingness to pay** | |
| --- | --- | --- | --- | --- | --- |
|  | **(*n*=1423)**  ***n* (%)** | **Mean (SD)** | ***p* value** | **Mean (SD)** | ***p* value** |
| **Spirituality** |  |  |  |  |  |
| Low (score <72) | 741 (52.1) | 3.68 (1.03) | <.001 | 2.64 (1.18) | .066 |
| High (score ≥72) | 682 (47.9) | 4.10 (0.90) |  | 2.52 (1.17) |  |
| **Health engagement (HE)**  I can manage my own health effectively. (HE1) |  |  |  |  |  |
| Disagree | 421 (29.6) | 3.00 (1.06) | <.001 | 2.26 (1.09) | <.001 |
| Agree | 1002 (70.4) | 4.25 (0.68) |  | 2.72 (1.18) |  |
| I spend a lot of time informing myself about health. (HE2) |  |  |  |  |  |
| Disagree | 515 (36.2) | 3.17 (1.10) | <.001 | 2.26 (1.07) | <.001 |
| Agree | 908 (63.8) | 4.29 (0.64) |  | 2.77 (1.19) |  |
| I can manage my own health even under stress. (HE3) |  |  |  |  |  |
| Disagree | 433 (30.4) | 3.01 (1.07) | <.001 | 2.27 (1.09) | <.001 |
| Agree | 990 (69.6) | 4.27 (0.65) |  | 2.72 (1.18) |  |
| I usually share concerns about my own health with my general practitioner. (HE4) |  |  |  |  |  |
| Disagree | 468 (32.9) | 3.07 (1.07) | <.001 | 2.22 (1.03) | <.001 |
| Agree | 955 (67.1) | 4.28 (0.65) |  | 2.77 (1.20) |  |
| I usually tell my general practitioner about unusual symptoms. (HE5) |  |  |  |  |  |
| Disagree | 485 (34.1) | 3.11 (1.07) | <.001 | 2.23 (1.06) | <.001 |
| Agree | 938 (65.9) | 4.28 (0.65) |  | 2.77 (1.19) |  |
| It is important to cooperate with healthcare workers in defining how to manage my own health. (H6) |  |  |  |  |  |
| Disagree | 358 (25.2) | 2.82 (1.04) | <.001 | 2.20 (1.05) | <.001 |
| Agree | 1065 (74.8) | 4.24 (0.67) |  | 2.72 (1.19) |  |
| **Attitudes towards vaccines (AVs)**  A vaccination could have serious collateral effects on my own health. (AVs1) |  |  |  |  |  |
| Disagree | 777 (54.6) | 3.49 (1.07) | <.001 | 2.42 (1.13) | <.001 |
| Agree | 646 (45.4) | 4.35 (0.61) |  | 2.78 (1.21) |  |
| I am sure of vaccines’ effectiveness in preventing infectious diseases. (AV2s) |  |  |  |  |  |
| Disagree | 525 (36.9) | 3.11 (1.02) | <.001 | 2.18 (1.05) | <.001 |
| Agree | 898 (63.1) | 4.33 (0.64) |  | 2.82 (1.18) |  |

Data are presented as the mean ± standard deviation (SD), frequency, and percentage. COVID-19 = coronavirus disease 2019; AVs = attitude towards vaccines; HE = health engagement. *p* values were calculated using an independent *t*-test; *p*<.05 indicates statistical significance.
