# Supplementary material for: Relationship of spirituality, health engagement, health belief and attitudes toward acceptance and willingness to pay for a COVID-19 vaccine
Source: PLoS One. 2022 Oct 12;17(10):e0274972. doi: 10.1371/journal.pone.0274972 (PMC9555617; doi:10.1371/journal.pone.0274972)
Supplement: S2 Table — (DOCX) [file pone.0274972.s004.docx]

**S2 Table. Comparisons of Citizen’s Health Beliefs with Their Acceptance and Willingness to Pay for COVID-19 Vaccine (*n*=1423)**

| **Variables** | **All participants** | **Acceptance** | | **Willingness to pay** | |
| --- | --- | --- | --- | --- | --- |
|  | **(*n*=1423)**  ***n* (%)** | **Mean (SD)** | ***p* value** | **Mean (SD)** | ***p* value** |
| **Health beliefs *–* Perceived susceptibility (PSU)**  My chance of getting COVID-19 in the next few months is great. (PSU1) |  |  |  |  |  |
| Disagree | 696 (48.9) | 3.60 (1.08) | <.001 | 2.32 (1.09) | <.001 |
| Agree | 726 (51.1) | 4.16 (0.81) |  | 2.84 (1.20) |  |
| I am worried about the likelihood of getting COVID-19 in the future. (PSU2) |  |  |  |  |  |
| Disagree | 632 (44.4) | 3.56 (1.11) | <.001 | 2.36 (1.13) | <.001 |
| Agree | 791 (55.6) | 4.14 (0.80) |  | 2.76 (1.18) |  |
| Getting COVID-19 is currently a possibility for me. (PSU3) |  |  |  |  |  |
| Disagree | 555 (39.0) | 3.51 (1.13) | <.001 | 2.35 (1.13) | <.001 |
| Agree | 868 (61.0) | 4.12 (0.81) |  | 2.74 (1.18) |  |
| **Health beliefs – Perceived severity (PSE)**  Complications from COVID-19 are serious. (PSE1) |  |  |  |  |  |
| Disagree | 694 (48.8) | 3.66 (1.08) | <.001 | 2.41 (1.13) | <.001 |
| Agree | 729 (51.2) | 4.09 (0.84) |  | 2.75 (1.19) |  |
| I will be very sick if I get COVID-19. (PSE2) |  |  |  |  |  |
| Disagree | 635 (44.6) | 3.60 (1.11) | <.001 | 2.39 (1.15) | <.001 |
| Agree | 788 (55.4) | 4.11 (0.82) |  | 2.74 (1.17) |  |
| I am afraid of getting COVID-19. (PSE3) |  |  |  |  |  |
| Disagree | 592 (41.6) | 3.51 (1.13) | <.001 | 2.35 (1.13) | <.001 |
| Agree | 831 (58.4) | 4.12 (0.81) |  | 2.74 (1.18) |  |
| **Health beliefs – Perceived benefits (PBE)**  Vaccination is a good idea because I feel less worried about catching COVID-19. (PBE1) |  |  |  |  |  |
| Disagree | 579 (40.7) | 3.51 (1.06) | <.001 | 2.24 (1.07) | <.001 |
| Agree | 844 (59.3) | 4.14 (0.85) |  | 2.82 (1.19) |  |
| Vaccination decreases my chances of getting COVID-19 and its complications. (PBE2) |  |  |  |  |  |
| Disagree | 573 (40.3) | 3.52 (1.05) | <.001 | 2.22 (1.03) | <.001 |
| Agree | 850 (59.7) | 4.13 (0.87) |  | 2.83 (1.20) |  |
| If I get vaccinated, I will decrease the frequency of having to consult my doctor. (PBE3) |  |  |  |  |  |
| Disagree | 1062 (74.6) | 3.80 (1.00) | <.001 | 2.48 (1.12) | <.001 |
| Agree | 361 (25.4) | 4.13 (0.92) |  | 2.89 (1.28) |  |
| **Health beliefs – Perceived barriers (PBA)**  The side-effects of vaccination may interfere with my usual activities. (PBA1) |  |  |  |  |  |
| Disagree | 1160 (81.5) | 3.97 (0.90) | <.001 | 2.58 (1.13) | .949 |
| Agree | 263 (18.5) | 3.48 (1.26) |  | 2.59 (1.35) |  |
| I am scared of needles. (PBA2) |  |  |  |  |  |
| Disagree | 1146 (80.5) | 4.00 (0.90) | <.001 | 2.58 (1.14) | .957 |
| Agree | 277 (19.5) | 3.40 (1.20) |  | 2.59 (1.32) |  |
| I cannot be bothered to get a vaccination. (PBA3) |  |  |  |  |  |
| Disagree | 952 (66.9) | 3.98 (0.90) | <.001 | 2.71 (1.11) | <.001 |
| Agree | 471 (33.1) | 3.68 (1.13) |  | 2.34 (1.26) |  |

Data are presented as the mean ± standard deviation (SD), frequency, and percentage. COVID-19 = coronavirus disease 2019; PBA = perceived barriers; PBE = perceived benefits; PSE = perceived severity; PSU = perceived susceptibility. *p* values were calculated using an independent *t*-test; *p*<0.05 indicates statistical significance.
