# Supplementary material for: Relationship of spirituality, health engagement, health belief and attitudes toward acceptance and willingness to pay for a COVID-19 vaccine
Source: PLoS One. 2022 Oct 12;17(10):e0274972. doi: 10.1371/journal.pone.0274972 (PMC9555617; doi:10.1371/journal.pone.0274972)
Supplement: S3 Table — (DOCX) [file pone.0274972.s005.docx]

**S3 Table. Adjusted Beta-Coefficients and 95% Confidence Intervals (CIs) of Spirituality, Health Engagement, and Attitude Toward Vaccine with Participants' Acceptance and Willingness to Pay for COVID-19 Vaccine (*n*=1423)**

| **Variable** | **Acceptance** | | **Willingness to pay** | |
| --- | --- | --- | --- | --- |
|  | **Unadjusted coef. β (95% CI)** | **Adjusted coef. β (95% CI)^a^** | **Unadjusted coef. β (95% CI)** | **Adjusted coef. β (95% CI)^b^** |
| **Spirituality** |  |  |  |  |
| Low (score <72) | Ref. | Ref. | Ref. | Ref. |
| High (score ≥72) | 0.43 (0.33~0.53)** | 0.14 (0.07~0.21)** | -0.11  (-0.24~0.01) | -0.25  (-0.37~-0.14)** |
| **Health engagement (HE)**  I can manage my own health effectively. (HE1) |  |  |  |  |
| Disagree | Ref. | Ref. | Ref. | Ref. |
| Agree | 1.25 (1.16~1.34)** | 0.20 (0.09~0.31)** | 0.46 (0.33~0.59)** | -0.01  (-0.18~0.18) |
| I spend a lot of time informing myself about health. (HE2) |  |  |  |  |
| Disagree | Ref. | Ref. | Ref. | Ref. |
| Agree | 1.11 (1.02~1.20)** | 0.13 (0.03~0.23)** | 0.50 (0.38~0.63)** | 0.12  (-0.04~0.28) |
| I can manage my own health even under stress. (HE3) |  |  |  |  |
| Disagree | Ref. | Ref. | Ref. | Ref. |
| Agree | 1.26 (1.17~1.35)** | 0.25 (0.14~0.35)** | 0.46 (0.33~0.59)** | 0.05  (-0.13~0.23) |
| I usually share concerns about my own health with my general practitioner. (HE4) |  |  |  |  |
| Disagree | Ref. | Ref. | Ref. | Ref. |
| Agree | 1.20 (1.11~1.29)** | 0.21 (0.10~0.31)** | 0.55 (0.42~0.68)** | 0.16  (-0.01~0.34) |
| I usually tell my general practitioner about unusual symptoms. (HE5) |  |  |  |  |
| Disagree | Ref. | Ref. | Ref. | Ref. |
| Agree | 1.17 (1.01~1.26)** | 0.11 (0.01~0.23)* | 0.53 (0.41~0.66)** | 0.13  (-0.05~0.30) |
| It is important to cooperate with healthcare workers in defining how to manage my own health. (H6) |  |  |  |  |
| Disagree | Ref. | Ref. | Ref. | Ref. |
| Agree | 1.41 (1.32~1.51)** | 0.28 (0.16~0.40)** | 0.52 (0.38~0.66)** | -0.06  (-0.27~0.14) |
| **Attitudes towards vaccines (AVs)**  A vaccination could have serious collateral effects on my own health. (AVs1) |  |  |  |  |
| Disagree | Ref. | Ref. | Ref. | Ref. |
| Agree | 0.86 (0.77~0.96)** | 0.29 (0.21~0.36)** | 0.35 (0.23~0.47)** | 0.04  (-0.09~0.17) |
| I am sure of vaccines’ effectiveness in preventing infectious diseases. (AVs2) |  |  |  |  |
| Disagree | Ref. | Ref. | Ref. | Ref. |
| Agree | 1.22 (1.14~1.31)** | 0.31 (0.21~0.41)** | 0.64 (0.52~0.76)** | 0.17 (0.01~0.33)* |

β = beta; COVID-19 = coronavirus disease 2019; CIs = confidence intervals; AVs = attitude towards vaccines; HE = health engagement. Adjusted beta-coefficients (coef.) and 95% CIs were estimated using a multiple linear regression after adjusting for ^a^ geographical region or ^b^ gender, age, income, geographical region, urbanicity, pandemic impact on income. * *p*<.05; ** *p*<.001.
