# Supplementary material for: Relationship of spirituality, health engagement, health belief and attitudes toward acceptance and willingness to pay for a COVID-19 vaccine
Source: PLoS One. 2022 Oct 12;17(10):e0274972. doi: 10.1371/journal.pone.0274972 (PMC9555617; doi:10.1371/journal.pone.0274972)
Supplement: S4 Table — (DOCX) [file pone.0274972.s006.docx]

**S4 Table. Adjusted Beta-coefficients and 95% Confidence Intervals (CIs) of Health Beliefs Constructs with Participants' Acceptance and Willingness to Pay for COVID-19 Vaccine (*n*=1423)**

| **Variable** | **Acceptance** | | **Willingness to pay** | |
| --- | --- | --- | --- | --- |
|  | **Unadjusted coef. β (95% CI)** | **Adjusted coef. β (95% CI)^a^** | **Unadjusted coef. β (95% CI)** | **Adjusted coef. β (95% CI)^b^** |
| **Health beliefs *–* Perceived susceptibility (PSU)**  My chance of getting COVID-19 in the next few months is great. (PSU1) |  |  |  |  |
| Disagree | Ref. | Ref. | Ref. | Ref. |
| Agree | 0.56 (0.46~0.66)** | 0.19 (0.06~0.32)** | 0.52 (0.40~0.64)** | 0.47 (0.25~0.68)** |
| I am worried about the likelihood of getting COVID-19 in the future. (PSU2) |  |  |  |  |
| Disagree | Ref. | Ref. | Ref. | Ref. |
| Agree | 0.58 (0.48~0.67)** | 0.05 (-0.10~0.19) | 0.40 (0.28~0.52)** | 0.01  (-0.24~0.24) |
| Getting COVID-19 is currently a possibility for me. (PSU3) |  |  |  |  |
| Disagree | Ref. | Ref. | Ref. | Ref. |
| Agree | 0.61 (0.50~0.71)** | -0.16 (-0.32~0.01) | 0.39 (0.27~0.52)** | -0.02  (-0.29~0.25) |
| **Health beliefs – Perceived severity (PSE)**  Complications from COVID-19 are serious. (PSE1) |  |  |  |  |
| Disagree | Ref. | Ref. | Ref. | Ref. |
| Agree | 0.43 (0.33~0.53)** | -0.08 (-0.20~0.04) | 0.35 (0.23~0.47)** | -0.18  (-0.38~0.02) |
| I will be very sick if I get COVID-19. (PSE2) |  |  |  |  |
| Disagree | Ref. | Ref. | Ref. | Ref. |
| Agree | 0.52 (0.42~0.62)** | -0.02 (-0.15~0.11) | 0.35 (0.23~0.48)** | 0.09  (-0.13~0.30) |
| I am afraid of getting COVID-19. (PSE3) |  |  |  |  |
| Disagree | Ref. | Ref. | Ref. | Ref. |
| Agree | 0.58 (0.48~0.68)** | 0.23 (0.08~0.38)** | 0.31 (0.18~0.43)** | -0.07  (-0.32~0.18) |
| **Health beliefs – Perceived benefits (PBE)**  Vaccination is a good idea because I feel less worried about catching COVID-19. (PBE1) |  |  |  |  |
| Disagree | Ref. | Ref. | Ref. | Ref. |
| Agree | 0.64 (0.54~0.74)** | 0.18 (0.07~0.28)** | 0.58 (0.45~0.70)** | 0.16  (-0.02~0.34) |
| Vaccination decreases my chance of getting COVID-19 or its complications. (PBE2) |  |  |  |  |
| Disagree | Ref. | Ref. | Ref. | Ref. |
| Agree | 0.61 (0.51~0.71)** | 0.03 (-0.09~0.14) | 0.62 (0.50~0.74)** | 0.24 (0.05~0.42)* |
| If I get vaccinated, I will decrease the frequency of having to consult my doctor. (PBE3) |  |  |  |  |
| Disagree | Ref. | Ref. | Ref. | Ref. |
| Agree | 0.33 (0.21~0.45)** | 0.10 (0.01~0.18)* | 0.41 (0.27~0.55)** | 0.13  (-0.01~0.27) |
| **Health beliefs – Perceived barriers (PBA)**  The side-effects of vaccination may interfere with my usual activities. (PBA1) |  |  |  |  |
| Disagree | Ref. | Ref. | Ref. | Ref. |
| Agree | -0.49 (-0.62~-0.36)** | -0.29 (-0.39~-0.19)** | 0.01 (-0.15~0.16) | -0.05  (-0.21~0.11) |
| I am scared of needles. (PBA2) |  |  |  |  |
| Disagree | Ref. | Ref. | Ref. | Ref. |
| Agree | -0.59 (-0.72~-0.47)** | -0.06 (-0.16~0.04) | 0.05 (-0.15~0.16) | 0.17 (0.01~0.34)* |
| I cannot be bothered to get a vaccination. (PBA3) |  |  |  |  |
| Disagree | Ref. | Ref. | Ref. | Ref. |
| Agree | -0.30 (-0.41~-0.19)** | -0.19 (-0.26~-0.11)** | -0.37 (-0.50~-0.24)** | -0.44  (-0.57~-0.32)** |

β = beta; CIs = confidence intervals; COVID-19 = coronavirus disease 2019; PBA = perceived barriers; PBE = perceived benefits; PSE = perceived severity; PSU = perceived susceptibility. Adjusted beta-coefficients (coef.) and 95% CIs were estimated using a multiple linear regression after adjusting for ^a^ geographical region or ^b^ gender, age, income, geographical region, urbanicity, pandemic impact on income. * *p*<.05; ** *p*<.001.
