# Supplementary material for: Relationship of spirituality, health engagement, health belief and attitudes toward acceptance and willingness to pay for a COVID-19 vaccine
Source: PLoS One. 2022 Oct 12;17(10):e0274972. doi: 10.1371/journal.pone.0274972 (PMC9555617; doi:10.1371/journal.pone.0274972)
Supplement: S1 Data Set — (DOC) [file pone.0274972.s007.doc]

<https://figshare.com/articles/dataset/S1_File_Acceptance_Willingness_Vaccine/20400603>
