# Supplementary material for: Relationship of spirituality, health engagement, health belief and attitudes toward acceptance and willingness to pay for a COVID-19 vaccine
Source: PLoS One. 2022 Oct 12;17(10):e0274972. doi: 10.1371/journal.pone.0274972 (PMC9555617; doi:10.1371/journal.pone.0274972)
Supplement: S1 File — (DOC) [file pone.0274972.s008.doc]

<https://figshare.com/articles/online_resource/The_Strengthening_the_Reporting_of_Observational_Studies_in_Epidemiology_STROBE_protocol/20424897/2>
